# Supplementary material for: Differential 5′-tRNA Fragment Expression in Circulating Preeclampsia Syncytiotrophoblast Vesicles Drives Macrophage Inflammation
Source: Hypertension. 2024 Feb 16;81(4):876–86. doi: 10.1161/HYPERTENSIONAHA.123.22292 (PMC10956686; doi:10.1161/HYPERTENSIONAHA.123.22292)
Supplement: Supplementary file 1 [file hyp-81-876-s001.doc]

Title

Differential 5'-tRNA Fragment Expression in Circulating Preeclampsia Syncytiotrophoblast Vesicles Drives Macrophage Inflammation

Authors

William Robert Cooke^1^*

Peiyong Jiang^2, 3^

Lu Ji^2, 3^

Jinyue Bai^2, 3^

Gabriel Davis Jones^1^

Y. M. Dennis Lo^2, 3^

Christopher Redman^1^

Manu Vatish^1^

**Corresponding author:* [*william.cooke@wrh.ox.ac.uk*](mailto:william.cooke@wrh.ox.ac.uk)

*Dr William Cooke*

*Nuffield Department of Women’s and Reproductive Health, University of Oxford*

*Level 3, Women’s Centre, John Radcliffe Hospital. Oxford, OX3 9DU, UK*

Affiliations

1) Nuffield Department of Women’s and Reproductive Health, University of Oxford, Oxford, UK

2) Centre for Novostics, Hong Kong Science Park, Pak Shek Kok, New Territories, Hong Kong SAR, China

3) Department of Chemical Pathology, The Chinese University of Hong Kong, Prince of Wales Hospital, Shatin, New Territories, Hong Kong SAR, China

Short title

Trophoblast 5’-tRNA Fragments in Preeclampsia

Supplemental Tables

*Table S1*

|  | **Normal (n=6)** | **Preeclampsia (n=8)** | **P value** |
| --- | --- | --- | --- |
| Median maternal age (IQR) – years | 34.5 (30.5-38.75) | 32 (30.3-37.5) | 0.59 |
| Median pre-pregnancy BMI (IQR) | 27 (20.8-33.8) | 28.3 (23.8-38) | 0.43 |
| First pregnancy – no. (%) | 0 (0) | 4 (50) | 0.08 |
| Diabetes – no. (%) | 0 (0) | 0 (0) | >0.99 |
| Essential hypertension – no. (%) | 0 (0) | 2 (25) | 0.47 |
| Current smoker – no. (%) | 0 (0) | 0 (0) | >0.99 |
| Male fetus – no. (%) | 4 (67) | 4 (50) | 0.63 |
| Median birthweight (IQR) – g | 3775 (3419-4090) | 1398 (1110-2270) | 0.001 |
| Median gestational age at delivery (IQR) – weeks | 39.2 (38.8-40) | 31.4 (30-35.3) | 0.0007 |
| Median highest recorded SBP (IQR) – mmHg | 132 (113-135) | 164 (140-178) | 0.003 |
| Median highest recorded DBP (IQR) – mmHg | 74 (64-75) | 108 (94-112) | 0.003 |

Characteristics of women who donated their placentas for dual-lobe perfusion, described using median/interquartile range (IQR) and compared using unpaired, two-sided Mann-Whitney tests; or described using number (no.) and percentage (%) and compared using two-sided Fisher’s exact tests.

*Table S2*

| **ID** | **A** | **B** | **C** |
| --- | --- | --- | --- |
| **Sequence** | TCCCTGGTGGT  CTAGTGGTTAG  GATTCGGCGCT | GCATTTGTGGT  TCAGTGGTAGA  ATTCTCGCCTG | CCCCTGTGGTC  TAGTGGTTAGG  ATTCGGCGCC |
| **Length** | 33 | 33 | 32 |
| **Mapping no indel** | tRF-Glu-CTC | tRF-Gly-GCC | Unmatch |
| **Mapping ≤2 indel** | tRF-Glu-CTC | tRF-Gly-GCC | tRF-Glu-CTC |
| **Mean PE count** | 52051 | 1159 | 965 |
| **SD PE count** | 18436 | 296 | 124 |
| **Mean N count** | 18405 | 534 | 530 |
| **SD N count** | 5423 | 193 | 95 |
| **Log2Fold Change** | -1.5 | -1.117 | -0.865 |
| **Adjusted p value** | 0.0054 | 0.0225 | 0.0058 |
| **Max monocyte count** | 204.5 | 6.2 | 0.9 |
| **Max erythrocyte count** | 43.3 | 0.8 | 0 |
| **Max platelet count** | 10 | 2 | 0 |

Median normalised expression of three target 5’-tRFs (labelled A, B and C for brevity) in placental EVs and maximum normalised expression of the same 5’-tRFs in blood-cell datasets. All count values represent reads per million (RPM).

*Table S3*

| **Sample** | **SRA ID** |
| --- | --- |
| Monocyte_1 | SRR6453428 |
| Monocyte_2 | SRR6453427 |
| Monocyte_3 | SRR6453426 |
| Monocyte_4 | SRR6453386 |
| Monocyte_5 | SRR6453387 |
| Monocyte_6 | SRR6453388 |
| Erythrocyte_1 | SRR1664893 |
| Erythrocyte_2 | SRR1664894 |
| Erythrocyte_3 | SRR1664895 |
| Erythrocyte_4 | SRR1664896 |
| Erythrocyte_5 | SRR1664897 |
| Platelet_1 | SRR10282877, SRR10282878 |
| Platelet_2 | SRR10282879, SRR10282880 |

Sequence read archive (SRA) identifiers for blood cell datasets

*Table S4*

| **Assay type** | **Manufacturer** | **Target** | **Identifier** |
| --- | --- | --- | --- |
| TaqMan™ gene expression assay | Applied Biosystems, USA | *TBP* | Hs00188166_m1 |
|  |  | *GAPDH* | Hs02758991_g1 |
|  |  | *ICAM1* | Hs00164932_m1 |
|  |  | *VCAM1* | Hs01003372_m1 |
|  |  | *NOS3* | Hs01574665_m1 |
|  |  | *TNF* | Hs00174128_m1 |
|  |  | *IL1B* | Hs01555410_m1 |
|  |  | *IL12B* | Hs01011518_m1 |
| TaqMan™ MicroRNA Assay | Applied Biosystems, USA | cel-miR-39 | 000200 |
|  |  | miR518 | 001156 |

Quantitative polymerase chain reaction assays used for RNA detection

*Table S5*

|  | **Normal (n=12)** | **Preeclampsia (n=14)** | **P value** |
| --- | --- | --- | --- |
| Maternal age in years – median (IQR) | 30 (28-33) | 30 (26-35) | 0.69 |
| Pre-pregnancy BMI – median (IQR) | 24 (21-27) | 23 (22-25) | 0.80 |
| First pregnancy – no. (%) | 6 (50) | 11 (79) | 0.22 |
| Diabetes – no. (%) | 0 (0) | 0 (0) | >0.99 |
| Essential hypertension – no. (%) | 0 (0) | 0 (0) | >0.99 |
| Current smoker – no. (%) | 2 (17) | 0 (0) | 0.22 |
| Male fetus – no. (%) | 7 (58) | 5 (36) | 0.43 |
| Gestational age at sample in weeks – median (IQR) | 31.9 (30-33.4) | 31.3 (28.8-32.7) | 0.64 |
| Birthweight in grams – median (IQR) | 3180 (2943-3291) | 1623 (1103-1766) | <0.0001 |
| Gestational age at delivery in weeks – median (IQR) | 39.1 (37.5-39.8) | 32.1 (29.7-34.2) | <0.0001 |
| Highest recorded SBP in mmHg – median (IQR) | 131 (120-134) | 180 (160-180) | <0.0001 |
| Highest recorded DBP in mmHg – median (IQR) | 74 (70-80) | 110 (98-115) | <0.0001 |
| Days from diagnosis to delivery – median (IQR) | N/A | 9 (6-18) | - |

Characteristics of women who donated blood samples, described using median and interquartile range (IQR) and compared using unpaired, two-sided Mann-Whitney tests; or described using number (no.) and percentage (%) and compared using two-sided Fisher’s exact tests.

Supplemental Figures

*Figure S1*


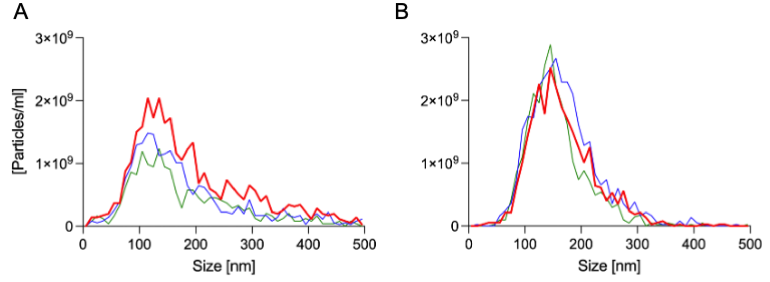


Nanoparticle Tracking Analysis size profiles for three separate samples for:

A) STB-MLEVs obtained by placental perfusion.

B) EVs obtained from eluate of membrane affinity columns loaded with maternal plasma.

*Figure S2*


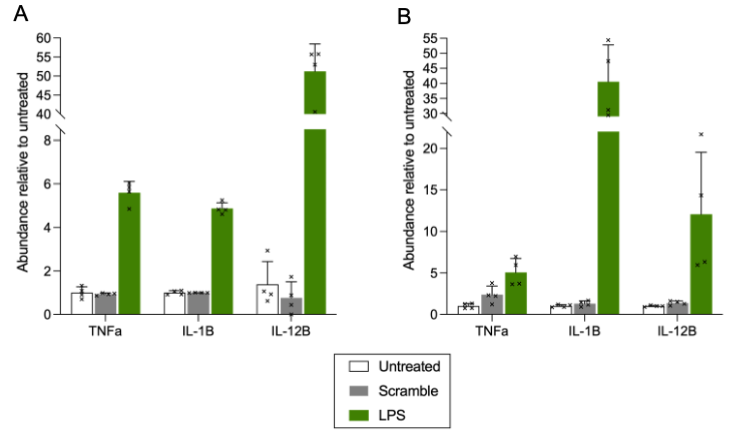


Scramble RNA control has no notable effect on macrophage cytokine expression when compared to untreated cells. Lipopolysaccharide (LPS) used as a positive control.
